# Effects of chicken manure substitution for mineral nitrogen fertilizer on crop yield and soil fertility in a reduced nitrogen input regime of North-Central China

**DOI:** 10.3389/fpls.2022.1050179

**Published:** 2022-12-15

**Authors:** Linyirui Ning, Xinpeng Xu, Yitao Zhang, Shicheng Zhao, Shaojun Qiu, Wencheng Ding, Guoyuan Zou, Ping He

**Affiliations:** ^1^ Key Laboratory of Plant Nutrition and Fertilizer, Ministry of Agriculture and Rural Affairs/Institute of Agricultural Resources and Regional Planning, Chinese Academy of Agricultural Sciences, Beijing, China; ^2^ Institute of Geographic Sciences and Natural Resources Research, Chinese Academy of Sciences, Beijing, China; ^3^ Institute of Plant Nutrition and Resources, Beijing Academy of Agriculture and Forestry Sciences, Beijing, China

**Keywords:** crop production, nutrient management, N recovery efficiency, soil C sequestration, soil organic matter

## Abstract

Organic manure has been proposed to substitute part of the chemical fertilizers. However, past research was usually conducted in regimes with excessive nitrogen (N) fertilization, which was not conducive to the current national goal of green and sustainable development. Therefore, exploring the potential of organic fertilizer substitution for mineral N fertilizer under regimes with reduced N inputs is important to further utilize organic fertilizer resources and establish sustainable nutrient management recommendations in the winter wheat (*Triticum aestivum* L.) – summer maize (*Zea mays* L.) rotation system in North-central China. In this study, a 4-year field experiment was conducted to investigate the effects of different chicken manure substitution ratios on crop yield, N recovery efficiency (REN), soil N and soil organic matter contents, to clarify the optimal organic substitution ratio of N fertilizer under reduced N application (from 540 kg N ha^−1^ year^−1^ to 400 kg N ha^−1^ year^−1^). Six substitution ratios were assessed: 0%, 20%, 40%, 60%, 80% and 100% under 200 kg N ha^−1^ per crop season, respectively, plus a control with no N application from chemical fertilizer or chicken manure. Results showed that the highest yield was achieved under the 20% substitution ratio treatment, with 1.1% and 2.3% higher yield than chemical N alone in wheat season and maize seasons, respectively. At the chicken manure substitution ratios of 20% in wheat season and 20%-40% in maize season, the highest REN reached to 31.2% and 26.1%, respectively. Chicken manure application reduced soil residual inorganic N with increasing substitution ratio. All organic substitution treatments increased soil organic matter and total N content. Implementing 20% organic substitution in wheat season and 20%-40% in maize season under the reduced N application regime in the North-central China is therefore recommended in order to achieve high crop yields and REN, improve soil fertility and enhance livestock manure resource utilization.

## 1 Introduction

As a large agricultural producer, China feeds 22% of the world’s population with 9% of its arable land (FAO, 2020). As a by-product of animal husbandry, a large amount of livestock and poultry manure has been generated. At present, China’s agriculture produces 5.7 billion tons of organic resources annually, of which livestock and poultry manure accounts for 66.7% (Niu and Ju, 2017). These manure resources are high-quality nutritional resources for agricultural production, but the utilization rate is less than 40% as reported by the Ministry of Agriculture and Rural Affairs of the People’s Republic of China (2017). Surveys showed that less than 10% of the arable land in China was applied with organic fertilizers (Chen et al., 2018). Therefore, improving the utilization rate of agricultural wastes and organic materials is a necessary way to achieve the development of green agriculture in the future.

Organic fertilizers are rich in nutrients and contain a variety of organic acids and peptides that help to improve soil fertility and crop yield (Valdez Velarca, 2016). For example, addition of exogenous carbon with organic fertilizers can promote the formation of soil aggregates and provide a favorable environment for microbial activities (Tripathi et al., 2014). Application of organic fertilizers can enrich mineralized N source and increase the soluble N content of the soil (Farrell et al., 2011; Wang et al., 2013), and the free amino acids produced by soil microbial decomposition microorganisms can be directly absorbed by crops (Näsholm et al., 2000). Compared with chemical fertilizers that quickly release nutrients into the soil, organic fertilizers can continuously supply nutrients, improve soil structure, promote nutrient cycling and transformation, and eventually enhance soil and crop productivity.

However, some disadvantages of organic fertilizers, such as a high volume for transportation and low nutrient contents resulting in slow crop response, lead farmers to focus more on chemical fertilization. To achieve high yields, chemical fertilizer application rate in China has increased 4.1 times over the past 40 years, with a 48% increase in N fertilizer (National Bureau of Statistics of China, 2020). This results in a series of problems, such as soil quality degradation, low nutrient use efficiency and serious environmental pollution (Galloway et al., 2008). One-third of the global total N pollution was caused by China (West et al., 2014). Using organic fertilizers to substitute part of the chemical fertilizers (hereafter called organic fertilizer substitution) has been suggested as an important measure to reduce chemical fertilizer inputs and mitigate their environmental consequences. Several previous studies have confirmed that long-term organic fertilizer substitution can improve soil fertility, and achieve high and stable crop yields compared to chemical fertilizer application only (Mi et al., 2016; Xie et al., 2016; Ding et al., 2018). In addition, organic fertilizer substitution was found to reduce soil inorganic N leaching (Elbl et al., 2020).

Improving fertilization techniques and reducing chemical N fertilizer application through combined organic-inorganic fertilization has become a pressing research topic in soil science and plant nutrition, as well as a trend for efficient use of agricultural resources in China’s agricultural development. The role of combined organic-inorganic fertilization in reducing chemical fertilizer application and increasing yields has been recognized (Zhang et al., 2018). However, many studies have focused on organic fertilizer substitution under high fertilizer application rates (Zhao et al., 2016; Xu et al., 2021), with limited research on reducing total nutrient input.

As an important grain production region in China, the North-central China has 18 million hectares of farmland, and its wheat and maize production is crucial to ensure national food security. Nevertheless, the N fertilizer application rate in this region is the highest among grain crops in China (Ju et al., 2009). The region is also a major livestock and poultry production region, for example with Handan as the second biggest city of chicken industry in China. Optimal fertilization is now receiving great attention as an essential measure to address the overuse of chemical fertilizers. In this context, chicken manure is a good alternative nutrient source for crop production, however, how to apply chicken fertilizer under reduced or optimized fertilization conditions needs further exploration. In this study, therefore, we investigated the effects of different ratios of chicken manure substitution under a regime of reduced N input in a winter wheat-summer maize rotation system in the North-central China. The objectives were to (1) assess the substitution effects on crop yield and N use efficiency, and on soil organic matter and soil N changes, and (2) identify the optimal ratio of chicken manure substitution.

## 2 Materials and methods

### 2.1 Study site and experimental design

A field experiment of winter wheat-summer maize rotation was conducted from June 2016 to June 2020 in Handan County, Hebei Province, North-central China (36°45’ 25’’ N, 115°19’ 21’’ E). The region has a temperate climate, and air temperature and precipitation were measured at an automatic meteorological station near the study site (**Supplementary Figure 1**). The soil type is fluvo-aquic soil, and the initial soil chemical properties at the 0-20 cm depth were: organic matter 1.83%, soil total N 0.12%, pH 8.56 (soil:water 1:2.5), Olsen-P 21.9 mg kg^−1^, and ammonium acetate (NH_4_OAc)-K 175 mg kg^−1^.

A fertilizer application survey with local farmers showed that N fertilizer application rate was up to 540 kg ha^−1^ year^−1^. In this study, the N application was reduced by 25% than farmers’ conventional rate, i.e., at 200 kg N ha^−1^ per crop season (400 kg ha^−1^ year^−1^). The experiment was set up with six different ratios of chicken manure substitution for chemical N fertilizer: 0%, 20%, 40%, 60%, 80% and 100%, which were noted as M0%, M20%, M40%, M60%, M80% and M100%, respectively. Moreover, a treatment with no N application (N0) was used as control (**Supplementary Table 1**). The field experiment employed a randomized block design with three replications, and each plot had an area of 30 m^2^. The amounts of P and K fertilizers applied were 33 kg P ha^−1^ and 75 kg K ha^−1^, respectively. The organic fertilizer used was chicken manure that had a pH 8.01 and contained 32.8% organic matter, 1.94% total N, 2.44% total P, and 1.45% total K on a dry matter basis. All fertilizers were applied as basal fertilizer in the maize season, and in two separate applications of 50%:50% during the basal and jointing stages in wheat season. Chicken manure was spread evenly on the soil surface and turned into the soil before planting each season. Other management measures for controlling weeds, pests, and diseases, were taken by spraying of herbicides (Tribenuron-methyl and 2.4-D for wheat; Atrazine and Propisochlor for maize) and insecticides (Carbendazim, Acephate, Omethoate, and Tradimefon for wheat; Carbofuran for maize) in accordance with the farmers’ conventional practices.

### 2.2 Sampling and chemical analysis

At harvest of maize, two rows of plants in the middle of each plot were collected to determine maize yield and converted to 15.5% of the standard moisture content for the final maize grain yield. Another five plants were collected from each plot and separated into straw and grain to determine the harvest index and straw weight. While for wheat, three representative 1 m × 1 m subplots in each plot were sampled to determine grain yield and converted to 13.5% of standard moisture content for the final wheat grain yield. Another sample of wheat plants in a 50 cm long row was collected randomly from each plot, and separated into straw and grain to determine the harvest index and straw weight.

The harvested straw and grain from subsamples were dried to constant weight at 60°C for 72 h to determine dry matter weight, and then ground and digested with H_2_SO_4_-H_2_O_2_ using a Kjeldahl method to determine N concentration for straw and grain. The soils from the 0-100cm depth at 20 cm per layer were sampled before sowing and after harvest in each plot to determine soil inorganic N concentrations (NO_3_-N+NH_4_-N) using flow injection analyzer (model AA3 -A001-02E, Brambleau, Germany). Soil water content was measured by oven drying the samples at 105°C. Soil total N and organic carbon were determined by the Kjedahl method and the dichromate oxidation method, respectively (Chinese Society of Soil Science, 2000).

### 2.3 Calculation and statistical analysis

The accumulated recovery efficiency (REN) of N fertilizer application (include chemical and organic N) was used to assess N use efficiency as follows:


(1) $${\text{REN}}_{i}=\frac{\sum_{i=1}^{n}\left(U_{Fi}-U_{CKi}\right)}{\sum_{i=1}^{n}F_{Ni}}$$

where *i* is the season (*i* = 1,2,…), with maize or wheat counted as one season separately in a rotation; *U_F_* and *U_CK_* represent the total N uptake (kg ha^−1^) in the aboveground biomass in the treatments with N application and control, respectively; and *F_N_* is the N fertilizer application rate (kg ha^−1^).

Soil organic carbon (SOC) storage (Mg ha^−1^) in the topsoil layer (0-20 cm depth) was calculated as:


(2) $$T=\frac{BD\times C\times h}{100}$$

where *T* is the SOC storage (Mg ha^−1^); *BD* is the soil bulk density (g cm^−3^); *C* is SOC content (g kg^−1^); and *h* (cm) is the soil thickness of top layer.

The amount of SOC sequestration (Mg ha^−1^) and the SOC sequestration rate (Mg ha^−1^ year^−1^) were calculated as follows:


(3) $$\text{SOC sequestration}=SOC_{f}-SOC_{i}$$

(4) $$SOC\;sequestration\;rate=\frac{Sequestration\;SOC}{year\;of\;experimentation}$$

where *SOC_f_* and *SOCi* are the SOC stocks in June 2020 and June 2016, respectively.

A one-way ANOVA analysis was conducted to evaluate the effects of chicken manure substitution ratio on maize and wheat yield, REN, soil total N and soil organic matter. Mean values of variables in each treatment were compared using Duncan’s multiple comparisons to identify significant differences among treatments at a significant level of *P*<0.05 using IBM SPSS Statistics 19 software (IBM Corp., Armonk, NY, USA). Graphs were plotted using the SigmaPlot software (SigmaPlot 14.0).

## 3 Results

### 3.1 Grain yield

All treatments with chicken manure substitution significantly increased wheat yield compared to N0 treatment (*P<0.001*). The M20% treatment obtained the highest average yield across all seasons with an average increase of 1.1% compared to the treatment with only chemical fertilizer (M0%). However, there were significant difference in wheat yields among the treatments with different substitution ratios. With increasing substitution ratios, wheat yields decreased by 1.5%, 2.6%, 8.9%, and 16.3% for M40%, M60%, M80%, and M100% treatments, respectively, compared to M0%. This indicated that high N fertilizer substitution ratios did not promote wheat yield under the reduced N input regime (**Table 1**).

**Table 1:** Yield of wheat and maize under treatments with different ratios of organic substitution for mineral N fertilizer.

Treatment	Wheat yield (kg ha^−1^)				Average yield(kg ha^−1^)	Yield increase rate (%)
	2016-2017	2017-2018	2018-2019	2019-2020		
N0	6388b	2493b	4505c	2693c	4020b	–
M0%	7641a	4098a	7599a	8819a	7039a	–
M20%	7696a	4005a	7748a	9003a	7113a	1.1
M40%	7570a	4314a	7433a	8416ab	6933a	-1.5
M60%	7663a	4118a	7279a	8362ab	6855a	-2.6
M80%	7327a	3728a	6449ab	8142ab	6412a	-8.9
M100%	7078ab	3487a	5491bc	7518b	5893a	-16.3
Treatment	Maize yield (kg ha^−1^)				Average yield (kg ha^−1^)	Yield increase rate (%)
	2016	2017	2018	2019
N0	8856a	7885b	6620b	6184c	7386c	–
M0%	9405a	9410a	8430a	8763ab	9002a	–
M20%	9279a	9916a	8489a	9146a	9207a	2.3
M40%	9575a	9486a	8352a	8695ab	9027a	0.3
M60%	9279a	9455a	8019a	8352ab	8777ab	-2.5
M80%	9415a	9204a	7904a	7506bc	8507ab	-5.5
M100%	8913a	9040a	7457ab	7355bc	8191b	-9.0

Yield increase rate is calculated as the yield difference between each treatment and M0% treatment divided by the yield of M0% treatment; different lowercase letters in the same column indicate significant differences between treatments under the same crop (P < 0.05).

For the maize season, although each treatment with chicken manure substitution increased maize yield compared to N0 treatment, maize yield remained high level without N application, reaching a 4-year average of 7386 kg ha^−1^ (**Table 1**). In terms of average maize yield across all seasons, the highest value was also observed in the M20% treatment, with a 2.3% increase compared to M0%. However, unlike wheat, the M40% and M0% treatments had similar yields. Although in 2016 and 2017 maize yields under the M40% treatment increased by 1.8% and 0.8%, respectively, compared to M0% treatment, the average maize yield increased by 0.3% across all seasons. In contrast, maize yields under the other chicken manure alternative treatments were lower than the M0% treatment, with reductions of 2.5%, 5.5% and 9.0% under M60%, M80% and M100% treatments, respectively.

The average annual yield of the wheat-maize rotation system showed a trend of increasing and then decreasing as the manure substitution ratio increased. This indicates that the higher percentage of chicken manure substitution for chemical fertilizer is not more effective in increasing crop yield. The results of this study showed that in the wheat-maize system, the M20% treatment had the highest average annual yield of 16,320 kg ha^−1^ with 25% less N application compared to farmers’ practices and the yield was 1.7% higher than that of M0%. In contrast, the lowest yield was observed in the M100% treatment with only 14,085 kg ha^−1^, which was 12.2% lower than that of M0%.

### 3.2 Nitrogen use efficiency

**Figure 1:**
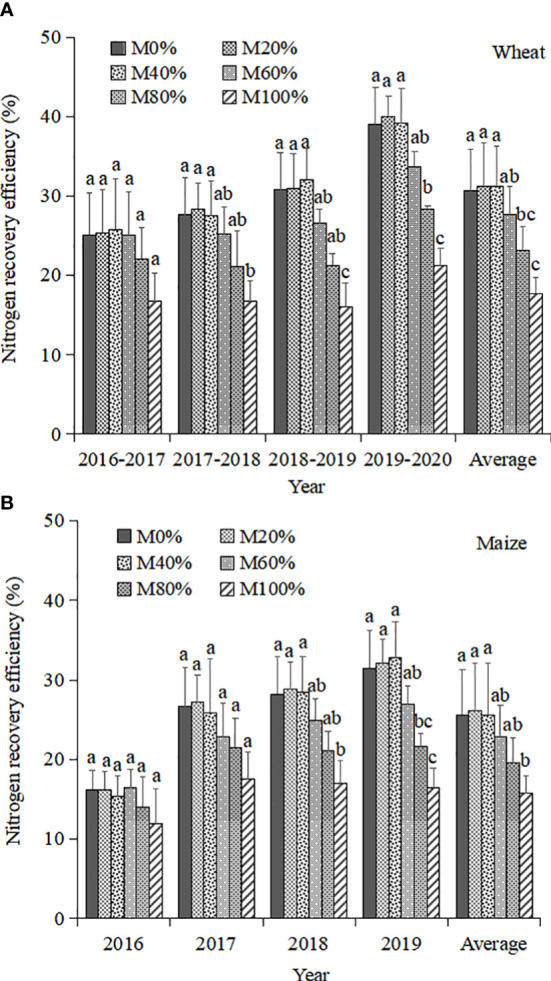
Nitrogen recovery efficiency of wheat **(A)** and maize **(B)** under treatments with different ratios of chicken manure substitution for mineral nitrogen fertilizer.

During the wheat season, REN tended to increase and then decrease with increasing chicken manure substitution ratio (**Figure 1A**). The REN reached the maximum at M40%, M20%, M40%, and M20% in the four wheat seasons, respectively. The highest REN of 31.2% was found both in the M20% and M40% treatments, which was 0.6% higher than in M0%, but the difference was not significant. The REN showed a decreasing trend when the chicken manure substitution ratio was greater than 60% and reached a significant reduction level when it was greater than 80%. Therefore, increasing the chicken manure substitution ratio of chemical N fertilizer under optimal N application rate did not result in a significant increase in REN, but an optimal substitution ratio exists to achieve highest yield.

The REN in the maize season followed the same trend as in the wheat season (**Figure 1B**). The highest REN was achieved at M60%, M20%, M20%, and M40% in the 4-year maize seasons, respectively. Across all maize seasons, on average, the highest REN was 26.1% and was achieved in the M20% treatment, which was 0.5% higher than the M0% treatment, but with no significant difference. A significant decrease in REN occurred when the chicken manure substitution ratio was greater than 40%. The reason for the lower REN in maize season compared to wheat season was that the experiment started in maize season, and thus the high initial soil N content resulted in a significantly lower REN in the first maize season (2016) than in other years.

The higher REN was attributed to the increase in N accumulation. Compared to M0%, higher N uptake could be maintained under M20% and M40% treatments, with the highest total N uptake across four-year under M20% treatment (**Supplementary Figure 2**). On the contrary, the crop N uptake gradually decreased when the substitution ratio was greater than 40%. Compared with the M0% treatment, the cumulative N uptake under the M100% treatment was 12.9% and 9.9% lower in the wheat and maize seasons, respectively (**Supplementary Figure 2**). This indicates that chemical N fertilizer is necessary to maintain soil N supply and meet crop nutrient requirements.

### 3.3 Soil nutrients and organic matter

**Figure 2:**
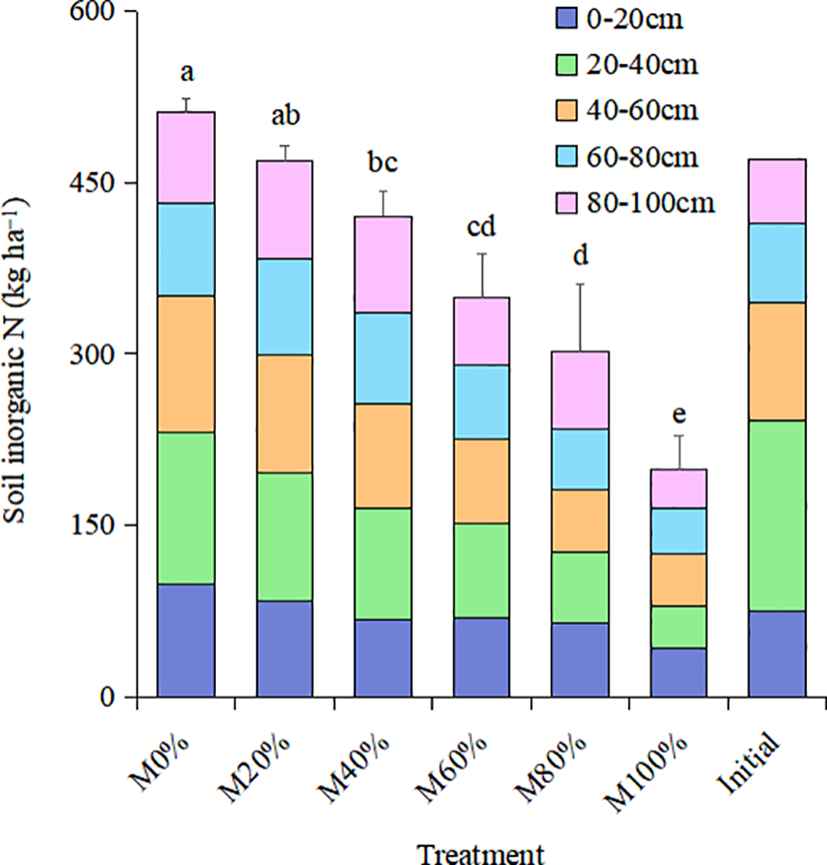
Soil inorganic nitrogen at harvest in 2020 at different soil layers with different organic substitution ratios of nitrogen fertilizer.

Soil residual mineral N at harvest showed a decreasing trend with increasing chicken manure substitution ratio (**Figure 2**). All substitution ratios reduced soil residual mineral N at harvest compared to M0% treatment, with the smallest reduction of 61.3% under M100% treatment. The mineral N content of the different soil layers also presented a tendency to decrease with increasing chicken manure substitution percentage, especially in the 0−60 cm depth. The treatments significantly reduced the mineral N content in the 20−40 cm soil layer compared to the initial soil, by 20.3%, 32.8%, 41.7%, 50.2%, 62.8%, and 78.0% under the M0%, M20%, M40%, M60%, M80%, and M100% treatments, respectively. This indicates that a reasonable chicken manure substitution ratio can reduce soil mineral N residues and prevent N leaching.

Chicken manure substitution for chemical N fertilizer reduced the soil mineral N content but increased soil total N content (**Table 2**). The soil total N content at each chicken manure substitution ratio ranged from 0.125% (M0%) to 0.163% (M100%), and was significantly higher than that of M0% treatment except for M20%. The soil total N content increased by 5.6%, 15.2%, 20.0%, 21.6%, and 30.4% in the treatment ranging from low to high chicken manure substitution ratios compared to M0% treatment.

**Table 2:** Soil organic matter (SOM), total N, Olsen-P and available K at harvest in 2020 for different organic substitution ratios of N fertilizer.

Treatment	SOM(%)	Total N(%)	Olsen-P(mg kg^−1^)	Available K(mg kg^−1^)
M0%	1.87 d	0.125 c	24.0 e	196.0 e
M20%	2.39 c	0.132 c	29.2 e	264.2 de
M40%	2.54 bc	0.144 b	38.0 d	356.1 cd
M60%	2.70 ab	0.150 b	44.7 c	389.9 bc
M80%	2.79 a	0.152 ab	53.1 b	457.4 ab
M100%	2.86 a	0.163 a	62.3 a	499.4 a

Different letters after the numbers in the same column indicate a significant difference (P < 0.05) among the treatments.

In addition, chicken manure substitution also increased the content of Olsen-P and available K in each treatment (**Table 2**), with similar trends to soil total N. Except for M20%, the soil Olsen-P and available K contents were significantly higher under each chicken manure substitution treatment compared to the chemical fertilizer treatment alone. As compared to M0%, the soil Olsen-P content increased by 21.6%, 58.3%, 86.3%, 121.3%, and 159.6%, and the soil available K content increased by 34.8%, 81.7%, 98.9%, 133.4%, and 154.8%, respectively, with an increase in chicken manure substitution ratio.

**Figure 3:**
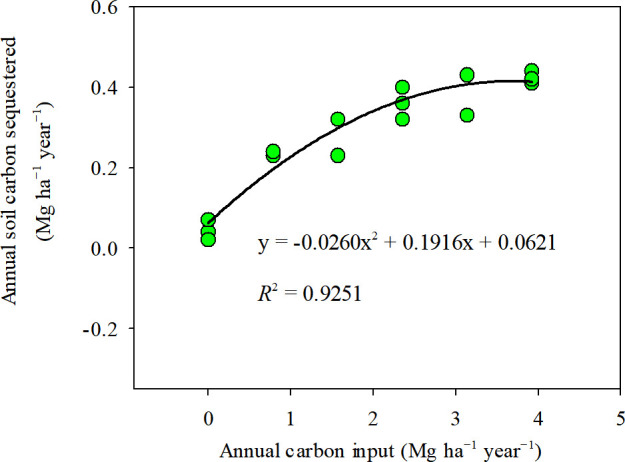
Relationship between annual soil organic carbon sequestration rate and annual carbon input in the wheat-maize rotation system.

Application of chicken manure significantly increased the soil organic matter content for each substitution ratio compared with M0% treatment, and the soil organic matter content increased with the increase in chicken manure substitution percentage (**Table 2**). The highest soil organic matter content of 2.86% was observed in the M100% treatment. All treatments with fertilizers increased soil organic matter content than the N0 treatment (1.77%), with the increase ranging from 5.4% in M0% to 61.2% in M100%. Compared with M0% (1.87%), the soil organic matter increased by 27.8%, 35.8%, 44.4%, 49.2%, and 52.9%, respectively, as the chicken manure substitution ratio ranged from 20% to 100%. Among the treatments, organic matter content was not significant between M20% (2.39%) and M40% (2.54%) treatments, and among M60% (2.70%), M80% (2.79%) and M100% (2.86%) treatments. The increase in soil organic matter was due to C inputs, and a significantly positive relationship was found between soil organic carbon (SOC) and C inputs (**Figure 3**). SOC sequestration rate per year increased with the increase of chicken manure substitution ratio in this study, from 0.23 Mg ha^−1^ year^−1^ in the M20% treatment to 0.42 Mg ha^−1^ year^−1^ in the M100% treatment.

### 3.4 Nitrogen reduction potential under reduced fertilizer application

**Figure 4:**
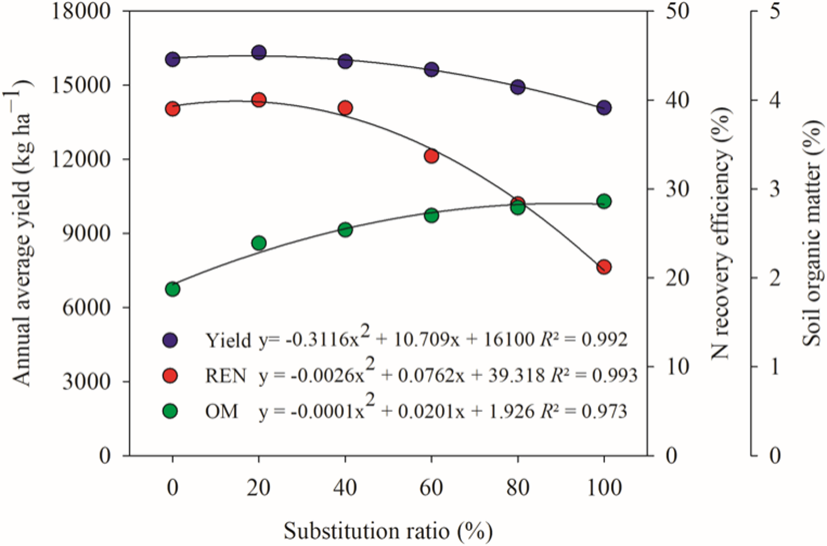
Relationship between different N fertilizer substitution ratios and average annual yield, N recovery efficiency (REN) and soil organic matter (OM) content in the wheat-maize rotation system.

From the average annual yield of the crop rotation system, crop yield decreased with the increase of the chicken manure substitution ratio, and only the M20% treatment showed a certain advantage in yield compared with conventional chemical fertilizer (M0%) by 1.7% increase (not significant). This indicated that the high chicken manure substitution ratio for chemical fertilizers played a negative effect on yield. Therefore, it is important to give a reasonable range of substitution under the reduced N input regime. The analysis of the relationship between the substitution ratio and the average annual yield, REN and soil organic matter content at the end of the experiment in the rotational system showed a significant quadratic curve (**Figure 4**). Using the highest yield obtained as a benchmark, the chicken manure substitution ratio in this study was 17.2% under the 200 kg N ha^−1^ per crop season, which was estimated to result in a yield of 16192 kg ha^−1^ year^−1^, accumulated REN of 39.9%, and an increase in the soil organic matter by 19.8%. However, the results obtained at this point were still lower than the performance of the M20% treatment, which was mainly limited by the number of treatments with low substitution ratios (below 20%). Therefore, in this study, considering the yield and REN, it is possible to replace 20% of chemical N fertilizer under the reduced N input regime. Of course, a reasonable chicken manure substitution ratio will be further increased with the continuous mineralization of the previous organic N inputs, and the current 20% substitution ratio is also the result of a 4-year field experiment.

## 4 Discussion

In the current nutrient management, N fertilizer still plays the greatest role in increasing yields in wheat and maize rotation systems. However, excessive N fertilizer application has caused negative impacts on soil and the environment. Organic fertilizer substitution, as one of the important measures for reducing N fertilizer, has played a positive role in chemical N fertilizer reduction and crop yield improvement (Yan et al., 2015). Organic fertilizer application not only enriches the soil with a large amount of nutrients, but also brings exogenous microorganisms into the soil (McGill et al., 1986), promotes the formation and decomposition of soil organic matter (Guo et al., 2016), accelerates nutrient cycling and contributes to crop yield increase (Han et al., 2021). Meanwhile, some small molecules from organic fertilizer application can serve to delay organ senescence in wheat and maize, prolong filling time, increase ear grain weight, and provide balanced nutrients for crops during later nutritional and reproductive growth, thus achieving yield increase (Redding et al., 2016; Sarkar et al., 2021).

Numerous studies have shown that organic fertilizers have great potential to reduce chemical N fertilizer and increase crop yields under current high N application. For example, organic fertilizer substitution of chemical N up to 50%-70% resulting in yield increase of 40.5% for maize (Xu et al., 2021), 23.3% for wheat (Li et al., 2022), 8.3% for early rice and 9.6% for late rice (Dai et al., 2021). However, most of these studies were conducted on organic fertilizer inputs under the premise of higher chemical N application, while with the implementation of various policies, limited standards and application of advanced technologies, N fertilizer use in China is on a decreasing trend (FAO, 2021)and will eventually return to reasonable levels. The study of organic substitution under reduced application is important for the sustainability of chemical fertilizer reduction. This study also showed that chemical N fertilizer can be replaced by 20% in wheat season and by 20%-40% in maize season with organic fertilizer under optimized N fertilizer application rate, meanwhile ensuring crop yield, which is consistent with some previous studies (Zhang et al., 2010; Duan et al., 2014). However, it has also been shown that organic fertilizers application with chemical fertilizers did not increase the yield of wheat and maize (Xin et al., 2017), and the main reason for this difference was that the N in organic fertilizer was not mineralized during the experimental period (Zhao et al., 2010). Compared to chemical fertilizers that have a fast-acting nature, a high ratio of organic fertilizer inputs would reduce crop nutrient uptake because of its slow nutrient release rate, resulting in inadequate supply of N in the early stage (Pang and Letey, 2000; Berry et al., 2002). In addition to chemical N input, the major part of soil available N source is from mineralization (Nyawade et al., 2020). As chicken manure is readily mineralized to an available inorganic N form, a combination of organic and inorganic applications provides a balanced and continuous supply of nutrients for the crop life cycle. Therefore, a certain amount of available nutrients at crop early growth period is necessary to maintain crop yield and support nutrient uptake (Paul et al., 2016).

The percentage of organic fertilizer substitution for chemical N fertilizer should be significantly lower under the regime of reduced fertilizer application compared to the regime of high chemical fertilizer input, otherwise it will negatively affect the crop yield. In this study, the yield of wheat−maize rotation showed a decreasing trend with increasing percentage of chicken manure substitution. However, the chicken manure substitution ratio in maize season can reach 40%, which is twice that of wheat season. The difference was mainly due to the increased mineralization of organic N by the simultaneous rain and heat in maize season.

It is indisputable that organic fertilizer application can increase soil organic matter (Kaur and Singh, 2014; Tripathi et al., 2014), and long-term application can serve to improve soil fertility. It is worth to note that a higher annual C addition with chicken manure produced a significant increase of sequestrated C by 0.19 Mg ha^−1^ year^−1^ in M20% to by 0.38 Mg ha^−1^ year^−1^ in M100% than M0% in this study. These findings indicated that the tested soils had great potential to sequestrate considerable amounts of C and build up SOC pool. The increase in SOC could be explained by the external C addition to the soil by manure application (Xu et al., 2023), because there was a positive relationship between SOC and C input (**Figure 3**), while the similar SOC content was observed between M0% and initial value. In addition, application of organic fertilizers exerts advantages in reducing soil bulk density (Bronick and Lal, 2005) and increasing soil porosity (Guo et al., 2016), and positively affects soil aggregation, macro porosity and water holding capacity, thus maintaining a high level of soil fertility (Petersen and Hoyle, 2016). Guo et al. (2016) showed that soil organic matter can be increased by 28-87% after 5 years of organic substitution under a wheat-maize rotation system. In addition, various nutrients in organic fertilizers complement the soil nutrient pool (Valdez Velarca, 2016; Guo et al., 2016), such as soil Olsen-P and available K contents (**Table 2**), and a good soil environment in turn promotes accumulation and hydrolysis of organic matter (Duan et al., 2017; Zhang et al., 2019; Liu et al., 2022). Various small molecule compounds in organic fertilizers also play a positive role in crop growth.

However, organic fertilizer is a double-edged sword for soil N. On the one hand, changes in the soil microenvironment can facilitate N cycling and transformation (He et al., 2015). On the other hand, N mineralization from organic fertilizer requires a time process that can negatively affect crop growth in the absence of available N (Choi et al., 2004; Guo et al., 2016). The greater C content in soil increases both nitrate immobilization and N demand (Liu et al., 2020). In this study, as the chicken manure substitution ratio increased, the soil total N content increased significantly by 1.4% in M20% to 7.6% in M100% compared to the chemical fertilizer application alone, but the soil mineral N content decreased significantly, which led to a significant decrease in crop yield. The study has shown that soil organic N content can be increased by up to 90% after organic fertilizer application (Rezig et al., 2013). The addition of organic fertilizer enhances $\text{N}{\text{H}}_{4}^{+}$ immobilization by slowly releasing mineral N from the decomposition processes, while facilitating the interaction between soil C and N cycles, thus significantly reducing N losses to the environment. Under the same N application rate in this study, the marked contrasting effects observed between total N and mineral N content suggested that manure input was favorable for N retention as complex organic forms (Tang et al., 2022). Therefore, replacing part of chemical fertilizer with manure is an effective method to reduce inorganic N surplus observed in soil of conventionally excessive chemical N fertilization. However, the organic N will be continuously mineralized along with the prolonged period of manure application, so the substitution ratio should also be increased. Further research and practice are still required to identify the optimal substitution ratio by considering long-term effects of manure applications.

The application of organic-inorganic fertilizers can decrease chemical N fertilizer inputs while reducing the instantaneous accumulation of inorganic N in the soil. Meanwhile, slow-release N from organic fertilizers reduces soil N losses while meeting nutrient requirements for later crop growth (Luo et al., 2011; Zhao et al., 2013). Conventional production patterns that rely on high amounts of chemical N fertilizer inputs not only lead to soil N accumulation and thereby increasing the risk of N leaching, but also increase the negative impacts on the ecosystem through ammonia volatilization and greenhouse gas emissions (Yao et al., 2013; Lassaletta et al., 2014; Wuepper et al., 2020). The recycling of organic resources such as livestock manure to farmland (Lassaletta et al., 2014; Bonaudo et al., 2014), i.e., partially replace chemical fertilizers, is one of the key strategies to mitigate soil nutrient losses (Elbl et al., 2020).

In recent years, studies on the application of organic materials in maintaining soil nutrient balance and reducing nutrient losses have been widely reported (Steiner et al., 2008; Beesley et al., 2011; Zhang et al., 2018). Tang et al. (2022) found that a 50% substitution with pig manure increased the diversity of bacteria, the relative abundance of specific microbiota involved in N cycling, the amount of denitrifiers, and the proportion of N_2_O-reducers, which resulted in a 44% reduction in agricultural inputs and less environmental impacts per unit of product. However, the reduction of fertilizer inputs and N losses should not be at the expense of crop yield, and organic fertilizer inputs should be kept at a reasonable range. This study showed that a 25% N fertilizer was reduced comparing with farmers’ conventional fertilizer application, and a further 20% chemical N fertilizer in wheat season and 20%-40% chemical N fertilizer in maize season can be replaced by chicken manure compared with the treatment of chemical fertilizer application alone (200 kg N ha^−1^ per season). This fertilization method would not only maintain crop yield but also reduce soil residual N while increasing N uptake. Liu et al. (2022) also showed that 30% organic substitution improved soil quality and increased maize yields. In the case of rice, application of 25%-50% organic fertilizer substitution could simultaneously increase yield and achieve agricultural sustainability (Song et al., 2022). These studies supported the results of our study. Therefore, adopting an appropriate organic substitution ratio under optimal fertilizer application conditions can effectively balance yield and environmental protection.

## 5 Conclusions

The results of a 4-year field experiment in the wheat-maize rotation of North-central China showed that partial substitution of chemical N fertilizer with chicken manure improved crop yield and N recovery efficiency, reduced soil mineral N residues, and significantly increased soil organic matter and total N content. Under the current chemical N application rate in this study (200 kg N ha^−1^ per season), organic substitutions by 20% in wheat season and 20%-40% in maize season were found to be suitable for the study area in terms of achieving high yield, N use efficiency and soil fertility. The study showed that manure fertilizer application increased soil Olsen-P and available K contents. However, the synergistic role of the manure application in optimizing the three macronutrients application should be explored in the future.

## Data availability statement

The original contributions presented in the study are included in the article/**Supplementary Material**. Further inquiries can be directed to the corresponding authors.

## Author contributions

LN analyzed the data and wrote the manuscript. XX designed the research and performed methodology. YZ and GZ participated in experimental research. SZ, SQ, and WD participated in data collation, investigation and validation. XX and PH commented on data interpretation and modified the manuscript. All authors contributed to the article and approved the submitted version.
