# Supplementary material for: Effects of chicken manure substitution for mineral nitrogen fertilizer on crop yield and soil fertility in a reduced nitrogen input regime of North-Central China
Source: Front Plant Sci. 2022 Dec 15;13:1050179. doi: 10.3389/fpls.2022.1050179 (PMC9798097; doi:10.3389/fpls.2022.1050179)
Supplement: Supplementary file 1 [file DataSheet_1.pdf]

Supplementary Material

Supplementary Table 1
The fertilizer application rate for each treatment per crop season.

Treatment	Chemical fertilizer (kg ha^{-1})			Chicken manure (kg ha^{-1})			
	N	P	K	N	P	K	C
M0%	200	33	75	0	0	0	0
M20%	160	33	75	40	50	30	392
M40%	120	33	75	80	100	60	785
M60%	80	33	75	120	150	90	1177
M80%	40	33	75	160	200	120	1569
M100%	0	33	75	200	250	150	1961
N0	0	33	75	0	0	0	0

Supplementary Figure 1

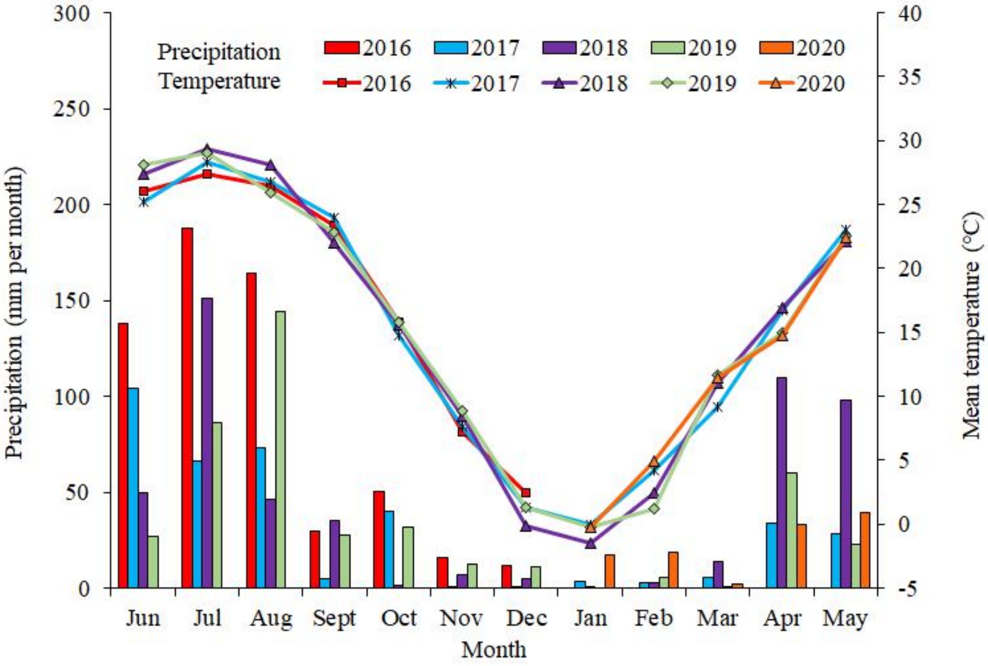

Supplementary Figure 1. Monthly mean air temperature and precipitation from June 2016 to June 2020 at the experimental winter wheat-summer maize rotation site in north-central China.

Supplementary Figure 2

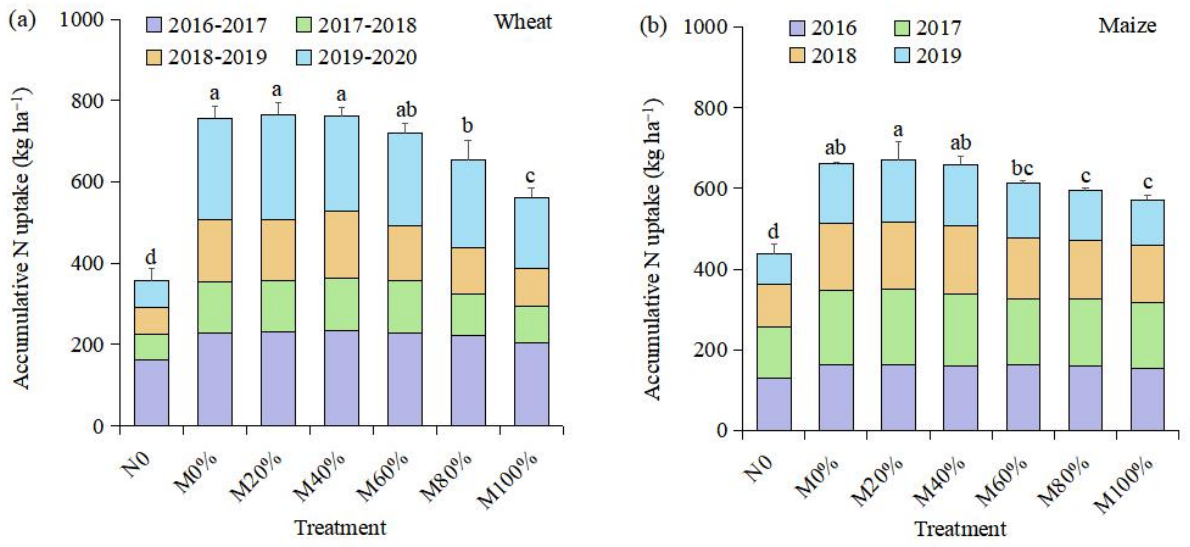

Supplementary Figure 2. Accumulative N uptake of wheat (a) and maize (b) under treatments with different ratios of chicken manure substitution for mineral nitrogen fertilizer.
